# PyBDA: a command line tool for automated analysis of big biological data sets

**DOI:** 10.1186/s12859-019-3087-8

**Published:** 2019-11-12

**Authors:** Simon Dirmeier, Mario Emmenlauer, Christoph Dehio, Niko Beerenwinkel

**Affiliations:** 1 Department of Biosystems Science and Engineering, ETH Zurich, Basel, Switzerland; 2 SIB Swiss Institute of Bioinformatics, Basel, Switzerland; 3 Biozentrum, University of Basel, Basel, Switzerland; 4 BioDataAnalysis GmbH, Munich, 81669 Germany

**Keywords:** Big data, Data analysis, Command line, Pipeline, Computing cluster, Grid engine, Machine learning

## Abstract

**Background:**

Analysing large and high-dimensional biological data sets poses significant computational difficulties for bioinformaticians due to lack of accessible tools that scale to hundreds of millions of data points.

**Results:**

We developed a novel machine learning command line tool called PyBDA for automated, distributed analysis of big biological data sets. By using Apache Spark in the backend, PyBDA scales to data sets beyond the size of current applications. It uses Snakemake in order to automatically schedule jobs to a high-performance computing cluster. We demonstrate the utility of the software by analyzing image-based RNA interference data of 150 million single cells.

**Conclusion:**

PyBDA allows automated, easy-to-use data analysis using common statistical methods and machine learning algorithms. It can be used with simple command line calls entirely making it accessible to a broad user base. PyBDA is available at https://pybda.rtfd.io.

## Background

The advent of technologies that produce very large amounts of high-dimensional biological data is posing not only statistical, but primarily computational difficulties for researchers in bioinformatics, including in single-cell sequencing, genome-wide association studies, or imaging [1–3]. For statistical analysis and machine learning of gene expression data, tools such as Scanpy [4] exist. However, they scale only up to a (few) million observations rendering them unsuitable for the analysis of, e.g., microscopy imaging data often comprising billions of cells. Approaches that scale to big data sets by using high-performance computing, such as reviewed in [5], have been developed mainly for sequence analysis, but not statistical analysis for data derived from, for instance, imaging or mass spectrometry.

Here, we introduce PyBDA, a Python command line tool for automated analysis of big biological data sets. PyBDA offers easily customizable machine learning pipelines that require only minimal programming knowledge. The main goal of PyBDA is to simplify the repetitive, time-consuming task of creating customized machine learning pipelines and combine it with distributed computation on high-performance clusters. The main contributions of PyBDA are (i) a command line tool for the analysis of big data sets with automated pipelines and generation of relevant plots after each analysis, (ii) various statistical and machine learning methods either using novel, custom implementations or interfacing to MLLib [6] from Apache Spark [7], and (iii) a modularized framework that can be easily extended to introduce new methods and algorithms. We built PyBDA with a special emphasis on ease of usability and automation of multiple machine learning tasks, such that minimal programming and implementation effort is required and tasks can be executed quickly.

## Overview

PyBDA provides various statistical methods and machine learning algorithms that scale to very large, high-dimensional data sets. Since most machine learning algorithms are computationally expensive and big high-dimensional data does not fit into the memory of standard desktop computers, PyBDA uses Apache Spark’s DataFrame API for computation which automatically partitions data across nodes of a computing cluster, or, if no cluster environment is available, uses the resources available.

In comparison to other data analysis libraries, for instance [8, 9], where the user needs to use the provided API, PyBDA is a command line tool that does not require extensive programming knowledge. Instead the user only needs to define a config file in which they specify the algorithms to be used. PyBDA then automatically builds a workflow and executes the specified methods one after another. PyBDA uses Snakemake [10] to automatically execute these workflows of methods.

**Fig. 1:**
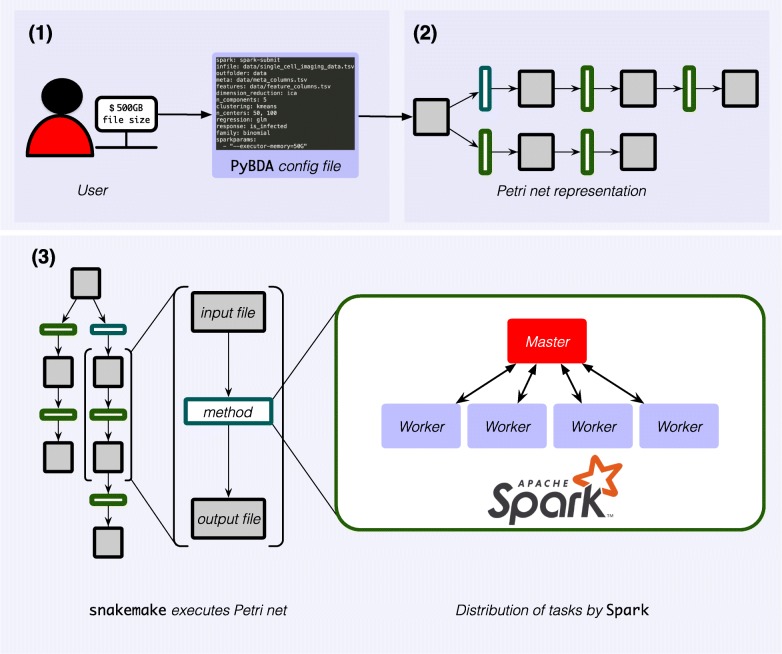
Using PyBDA. (1) To use PyBDA, the user only requires to create a short config file that lists the different methods to be executed. (2) From the config file, PyBDA creates an abstract Petri net, i.e., a bipartite directed graph with *data nodes* (gray squares) and *operation nodes* (analysis methods, green rectangles). (3) PyBDA traverses the net and creates triples, i.e., subgraphs consisting of an input file, an associated analysis method, and an output file. It then uses Snakemake for execution of each triple. The associated method of every triple is implemented as a Python module, each developed against the DataFrame API from Apache Spark. Spark uses a master to chunk a method into several tasks and distributes these on worker nodes on a distributed HPC cluster

Specifically, PyBDA implements the following workflow to enable pipelining of multiple data analysis tasks (Fig. 1): PyBDA builds an abstract Petri net from a config file containing a list of statistical methods or machine learning algorithms to be executed. A Petri net is a bipartite, directed graph in which one set of nodes represents conditions (in our case data sets) and the other set represents transitions (in our case operations like machine learning methods and statistical models). A transition in a Petri net model can only be enabled if a condition is met, i.e., in our case when a data set that is used as input for a method exists on the file system. Firing a transition leads to the creation of a new condition, i.e., a new data set. Every operation in the Petri net, i.e., every triple of input file, method and output file, is then executed by Snakemake. The method of every triple is a Python module with the main functionality being implemented with Spark’s DataFrame and RDD API or MLLib. By using Spark, data sets are automatically chunked into smaller pieces, and executed on a distributed high performance computing (HPC) cluster in parallel on multiple cores. Through distributed, parallel computing it is possible to fit models and apply methods even to big, high-dimensional data sets.

### Comparison to other big data tools

In the last decade several big data analysis and machine learning frameworks have been proposed, yet none of them allow for easy, automated pipelining of multiple data analysis or machine learning tasks. Here, we briefly compare the pros and cons of PyBDA with some of the most popular frameworks, including TensorFlow [11], scikit-learn [8], mlr [9], MLLib [6] and h20 [12]. Furthermore, many other machine learning tools, such as PyTorch [13], Keras [14] or Edward [15] that are comparable in functionality to the previous frameworks exist. For the sake of completeness, we also mention tools for probabilistic modelling, such as PyMC3 [16], GPFlow [17] or greta [18] which, of course, are primarily designed for statistical modelling and probabilistic programming and not for big data analysis.

We compare the different tools using the following criteria (Table 1): (1) how easily can the tool be used, especially w.r.t. programming knowledge (*usability*), (2) how much time does it take to implement a method/model once the API has been learned (*time to implement*), (3) how much knowledge of machine learning (ML), optimization, modelling and statistics is needed to use the tool (*ML knowledge*), (4) is it possible to use big data with the tool, i.e., does it scale well to big and high-dimensional data sets (*big data*), (5) how many methods are supported from scratch without the need to implement them (*supported methods*), and (6) is the tool easily extended with new methods, e.g., using the provided API (*extensibility*).

**Table 1:** Common statistical analysis and machine learning tools

	

We compare every tool by trichotomized criteria to evaluate if it places above average (green mark), on average (orange mark), or below average (red marks)

In comparison to PyBDA, the other methods we considered here are either complex to learn, take some time to get used to, or are not able to cope with big data sets. For instance, TensorFlow scales well to big, high-dimensional data sets and allows for the implementation of basically any numerical method. However, while being the most advanced of the compared tools, it has a huge, complex API and needs extensive knowledge of machine learning to be usable, for instance to implement the evidence lower bound of a variational autoencoder or to choose an optimizer for minimizing a custom loss function. On the other hand, tools such as scikit-learn and mlr are easy to use and have a large range of supported methods, but do not scale well, because some of their functionality is not distributable on HPC clusters and consequently not suitable for big data. The two tools that are specifically designed for big data, namely MLLib and h20, are very similar to each other. A drawback of both is the fact that the range of models and algorithms is rather limited in comparison to tools such as scikit-learn and mlr. In comparison to h20’s H20Frame API, we think Spark not only provides a superior DataFrame/RDD API that has more capabilities and is easier for extending a code base with new methods, but also has better integration for linear algebra. For instance, computation of basic descriptive statistics using map-reduce or matrix multiplication are easier implemented using Spark.

PyBDA is the only specifically built to not require much knowledge of programming or machine learning. It can be used right away without much time to get used to an API. Furthermore, due to using Spark it scales well and can be extended easily.

## Implementation

### Supported algorithms

PyBDA comes with a variety of algorithms for analysing big data from which the user can choose (Table 2). Unless already provided by MLLib, we implemented the algorithms against Spark’s DataFrame API. Especially efficient implementations of common scalable dimension reduction methods included in PyBDA, such as kernel principal component analysis (kPCA), independent component analysis (ICA), linear discriminant analysis (LDA) and factor analysis (FA), have been missing in current open source software entirely. PyBDA primarily supports simple models that do not trade biological interpretability for mathematical complexity and performance.

**Table 2:** Methods provided by PyBDA

Category	Method	Implementation
Dimension reduction	PCA	MLLib
	Factor analysis	custom implementation
	*k*-PCA	custom implementation
	Linear discriminant analysis	custom implementation
	Independent component analysis	custom implementation
Clustering	*k*-means	MLLib
	Gaussian mixture models	MLLib
Supervised learning	Random forests	MLLib
	Gradient boosting	MLLib
	Generalized linear models	MLLib

### Running pyBDA

**Fig. 2:**
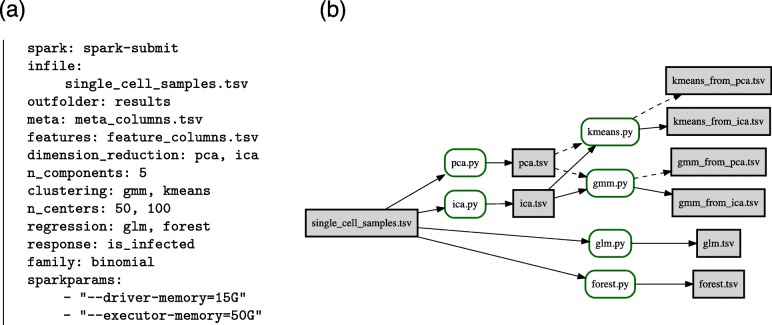
A PyBDA config file and the corresponding Petri net. Executing a config file (**a**) generates a corresponding Petri net (**b**). Here, PyBDA uses a file of single cells as input and then executes dimension reductions (PCA, ICA) and regression models (GLM, RF). The outputs from the dimension reductions are further used for clustering (GMM, *k*-means) resulting in a total of six sets of files

In order to run PyBDA on a Spark cluster, the user needs to provide an IP address to which Spark sends its jobs. Consequently, users need to either setup a cluster (standalone, Kubernetes, etc.) or submit jobs to the local host, where the strength of PyBDA is computation on a distributed HPC environment. Given the IP of the Spark cluster, the user needs to provide a config file with methods, data files, and parameterization. For instance, the config file provided in Fig. 2a will first trigger dimension reductions using principal component analysis (PCA) and ICA to 5 dimensions on a data set called single_cell_samples.tsv and feature names provided in feature_columns.tsv. PyBDA then uses the outputs of both methods and fits Gaussian mixture models (GMM) and runs *k*-means to each output with 50, or 100, cluster centers, respectively (resulting in four different results). In addition, a generalized linear model (GLM) and a random forest (RF) with binomial response variable (named is_infected) will be fitted on the same features. Thus, PyBDA automatically parses all combinations of methods and automatically executes each combination (Fig. 2b shows the corresponding Petri net of files and operations). The results of all methods are written to a folder called results. For each job, PyBDA allows Spark to use 15Gb of driver memory (for the master) and 50Gb memory for each executor (the main process run by a worker node).

## Results

**Fig. 3:**
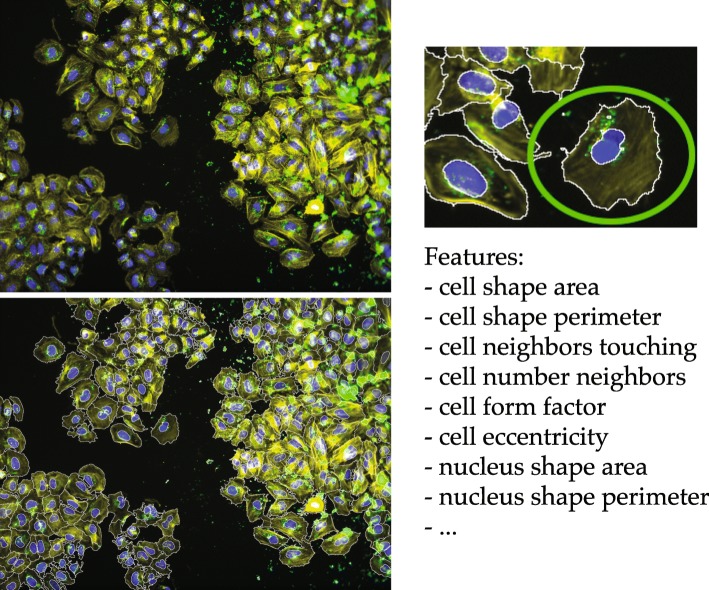
An example of a single-cell image used for segmentation and extraction of image features. We extracted image features of 150 million individual cells, their nuclei and their perinuclei. Cell bodies are stained in yellow, nuclei in blue, pathogens in green (left image). Features consist of cell, nucleus and perinuclei properties and information about local cell neighborhood, and if a cell is infected with a pathogen or not (right image)

In order to demonstrate PyBDA’s capability to deal with big biological data, we preprocessed the features extracted from microscopy imaging data of a large-scale RNA interference screen of the pathogen *B. henselae* and used them for big data analysis. In summary, HeLa cells have first been seeded on 384-well plates. In every well, a single gene has been knocked down and subsequently infected with *B. henselae*. After infection, images of cells have been taken for every plate and well, and for each cell, 43 image features have been extracted (Fig. 3). Features consist either of spatial/geometrical cell and nucleus properties (cells stained yellow, nuclei stained blue) or information about local cell neighborhood (Additional file 1 – features). Assuming that image features impact the cell’s infection we regressed the binary response of infection status on these features. Specifically, we hypothesized that cells in densely populated regions, or with comparatively little cell area, should on average be less vulnerable to infection in comparison to larger cells in sparsely populated regions (*B. henselae* stained green). Inference of the parameters for the infection status is of particular interest, because it could make using dedicated flourescence markers for pathogens obsolete. Since the data set consists of roughly 150 million single cells, a conventional analysis on desktop computers is not possible. However, it becomes feasible on a distributed environment using our command line tool PyBDA. Using a config file similar to the one in Fig. 2, we fit a generalized linear model with a binomial response, a random forest, and gradient boosting machines (GBM) to the data set. In order to avoid bias, PyBDA automatically balances the data set to contain equal fractions of each class by downsampling. We found that all three methods are capable of predicting the infection state of a cell from the image features well. Overall, the GLM performed slightly poorer (precision 0.70, recall 0.68) than the GBM (precision 0.73, recall 0.74; trained with 20 decision trees; subsampling rate of data 0.25) or the RF (precision 0.71, recall 0.71; same parameters). Since we are in an almost asymptotic regime of sample size *n*, splitting the data into train and test sets yields the same errors on both sets. Thus we are reporting the performance measures and parameters on the full data set here. While the RF and GBM improve performance, their biological interpretation is more challenging, because they do not establish simple, parametric dependencies as the GLM. For the GLM we found that features such as the cell area (*β*=0.21) or cell perimeter (*β*=0.18) contribute to enhanced infection, while features such as the number of cell neighbors (*β*=−0.11) decrease infectivity. Fitting the GLM required 2:30h runtime on an HPC platform, using a rather small cluster with two nodes and five cores each and 15 Gb of memory per core. Fitting the RF and the GBM took roughly 8h each, and required increasing the resources to five worker nodes with 10 cores and 20Gb each. The amount of parallelization and available computing resources is pivotal for runtime and insofar independent of PyBDA, as all computations are run by Spark. Runtime benchmarks of big data tools including Spark have, for instance, already been conducted by others [19, 20].

## Conclusion

PyBDA is a command line tool for machine learning of big biological data sets scaling up to hundreds of millions of data points. PyBDA automatically parses a user defined pipeline of multiple machine learning and data analysis tasks from a config file and distributes jobs to compute nodes using Snakemake and Apache Spark. We believe PyBDA will be a valuable and user-friendly tool supporting big data analytics and continued community-driven development of new algorithms.

## Availability and requirements

**Project name:** PyBDA

**Project home page:** https://github.com/cbg-ethz/pybda

**Operating system(s):** Linux and MacOS X

**Programming language:** Python

**Other requirements:** Python 3.6, Java JDK 8, Apache Spark 2.4.0

**License:** GNU GPLv3

**Any restrictions to use by non-academics:** License needed

## Supplementary information

**Additional file 1** Features used for the regression tasks.

## Data Availability

PyBDA is available on GitHub (https://github.com/cbg-ethz/pybda), the Python Package Index (https://pypi.org/project/pybda/), or Bioconda (https://bioconda.github.io/recipes/pybda/README.html). Documentation is available at https://pybda.readthedocs.io/en/latest/. The datasets used for the example are available from 10.3929/ethz-b-000360833.

## Abbreviations

FA: Factor analysis
GBM: Gradient boosting machines
GLM: Generalized linear model
GMM: Gaussian mixture model
HPC: High performance computing
ICA: Independent component analysis
LDA: Linear discriminant analysis
ML: Machine learning
PCA: Principal component analysis
RF: Random forest

## References

[1] Bühlmann P, van de Geer S (2018). Statistics for big data: A perspective. Stat Probab Lett.

[2] Katal A, Wazid M, Goudar RH. Big Data: Issues, Challenges, Tools and Good Practices. In: 2013 Sixth International Conference on Contemporary Computing (IC3). IEEE: 2013. p. 404–9. 10.1109/IC3.2013.661222.

[3] Marx V. The big challenges of big data. Nature 498. 2013. 10.1038/498255a 23765498

[4] Wolf FA, Angerer P, Theis FJ (2018). Scanpy: large-scale single-cell gene expression data analysis. Genome Biol.

[5] Guo R, Zhao Y, Zou Q, Fang X, Peng S (2018). Bioinformatics applications on Apache Spark. GigaScience.

[6] Meng X, Bradley J, Yavuz B, Sparks E, Venkataraman S, Liu D, Freeman J, Tsai D, Amde M, Owen S (2016). Mllib: Machine learning in Apache Spark. J Mach Learn Res.

[7] Zaharia M, Xin RS, Wendell P, Das T, Armbrust M, Dave A, Meng X, Rosen J, Venkataraman S, Franklin MJ (2016). Apache Spark: A unified engine for big data processing. Commun ACM.

[8] Pedregosa F, Varoquaux G, Gramfort A, Michel V, Thirion B, Grisel O, Blondel M, Prettenhofer P, Weiss R, Dubourg V (2011). Scikit-learn: Machine learning in Python. J Mach Learn Res.

[9] Bischl B, Lang M, Kotthoff L, Schiffner J, Richter J, Studerus E, Casalicchio G, Jones ZM (2016). mlr: Machine Learning in R. J Mach Learn Res.

[10] Köster J, Rahmann S (2012). Snakemake—a scalable bioinformatics workflow engine. Bioinformatics.

[11] Abadi M, Agarwal A, Barham P, Brevdo E, Citro C, Corrado GS, Davis A, Dean J, Devin M, et al.TensorFlow: Large-Scale Machine Learning on Heterogeneous Distributed Systems. arXiv preprint arXiv:1603.04467. 2016.

[12] H, 2O.ai. Python Interface for H2O. 2019. Python module version 3.26.0.2. https://github.com/h2oai/h2o-3.

[13] Paszke A, Gross S, Chintala S, Chanan G, Yang E, DeVito Z, Lin Z, Desmaison A, Antiga L, Lerer A. Automatic Differentiation in PyTorch. In: NIPS Autodiff Workshop: 2017.

[14] Chollet F, et al.Keras. 2015. https://keras.io.

[15] Tran D, Kucukelbir A, Dieng AB, Rudolph M, Liang D, Blei DM. Edward: A library for probabilistic modeling, inference, and criticism. arXiv preprint arXiv:1610.09787. 2016.

[16] Salvatier J, Wiecki TV, Fonnesbeck C (2016). Probabilistic programming in Python using PyMC3. PeerJ Comput Sci.

[17] Matthews DG, Alexander G, Van Der Wilk M, Nickson T, Fujii K, Boukouvalas A, León-Villagrá P, Ghahramani Z, Hensman J (2017). GPflow: A Gaussian Process Library using TensorFlow. J Mach Learn Res.

[18] Golding N. Greta: Simple and Scalable Statistical Modelling in R. 2018. R package version 0.3.0. https://CRAN.R-project.org/package=greta.

[19] Pafka S. benchm-ml. GitHub. 2019. https://github.com/szilard/benchm-ml/tree/941dfd4ebab3854b3a49fd70c192ecf21e483267.

[20] García-Gil D, Ramírez-Gallego S, García S, Herrera F (2017). A comparison on scalability for batch big data processing on Apache Spark and Apache Flink. Big Data Analytics.

